# A new genus and species of dalodesmid millipede from New South Wales, Australia (Diplopoda, Polydesmida, Dalodesmidae)

**DOI:** 10.3897/zookeys.517.10187

**Published:** 2015-08-13

**Authors:** Robert Mesibov

**Affiliations:** 1 Queen Victoria Museum and Art Gallery, 2 Invermay Road, Launceston, Tasmania 7248, Australia

**Keywords:** Diplopoda, Polydesmida, Dalodesmidae, New South Wales, Australia

## Abstract

*Cernethia inopinata* **gen. n.**, **sp. n.** is described from highland New South Wales. Like other dalodesmids the new species has numerous sphaerotrichomes on the legs of adult males, but *Cernethia inopinata* sp. n. shares several character states with Tasmanian species in the genera *Noteremus* Mesibov, 2009, *Paredrodesmus* Mesibov, 2003 and *Procophorella* Mesibov, 2003, which lack sphaerotrichomes and have not yet been assigned to family within the suborder Dalodesmoidea.

## Introduction

When establishing the new genus *Noteremus* from Tasmania (Mesibov 2009), it was suggested that *Noteremus* and two other dalodesmoid Tasmanian genera, *Paredrodesmus* Mesibov, 2003 and *Procophorella* Mesibov, 2003, might form a natural group. Species in all three genera have a head+19 rings body plan, reduced paranota (absent in *Paredrodesmus*), the unusual pore formula 5+7-18, a trapezoidal array of spinnerets (ventral pair further apart than dorsal pair) and no sphaerotrichomes on the legs of adult males. The lack of sphaerotrichomes led to the place ment of the three genera in the suborder Dalodesmidea without a family assignment.

Here a new species from highland New South Wales is described, which shares the first four of those character states, but unexpectedly has sphaerotrichomes on the tarsus and tibia of most legs. The gonopod conformation is also distinctive, and the telopodite lacks the clusters of stout, rod-like setae found in the two named *Noteremus* species and five of the six named *Paredrodesmus* species.

The new species is placed in a new genus in Dalodesmidae. The relationships of the new genus, *Noteremus*, *Paredrodesmus* and *Procophorella* to each other and to more typical Australian Dalodesmidae remain a puzzle.

## Materials and methods

‘Male’ and ‘female’ in the text refer to adult (stadium 7) individuals. Body measurements were estimated with a Nikon SMZ800 binocular dissecting microscope using an eyepiece scale. Colour images were manually stacked using a Canon EOS 1000D digital SLR camera mounted on the Nikon SMZ800 fitted with a beam splitter, then processed with Zerene Stacker 1.04. Gonopods were cleared in 80% lactic acid and temporarily mounted in a 1:1 glycerol:water mixture for optical microscopy. Preliminary gonopod drawings were traced from prints of screenshots from the output of a 1.3 megapixel digital video eyepiece camera mounted in one ocular tube of a Tasco LMSMB binocular microscope. Images and drawings were prepared for publication using GIMP 2.8.

Locality details for specimen lots (also available online in Mesibov 2006–2015) are given with latitude and longitude converted to decimal degrees based on the WGS84 datum. My estimate of the uncertainty for a locality is the radius of a circle around the given position, in metres or kilometres. The ANIC georeferences come from the ANIC collection database.

Abbreviations: AM = Australian Museum, Sydney, Australia; ANIC = Australian National Insect Collection, Canberra, Australia; NSW = New South Wales, Australia.

## Results

### Order Polydesmida Pocock, 1887 Suborder Dalodesmidea Hoffman, 1980 Family Dalodesmidae Cook, 1896

#### Genus Cernethia

Taxon classificationAnimaliaPolydesmidaDalodesmidae

Mesibov gen. n.

http://zoobank.org/09602805-528E-411A-9C96-B2EB11037259

##### Type species.

*Cernethia inopinata* sp. n., by present designation.

##### Other assigned species.

None.

##### Diagnosis.

Superficially resembling *Atalopharetra* Mesibov, 2005, *Bromodesmus* Mesibov, 2004 and *Victoriombrus* Mesibov, 2004 in having reduced paranota and an acrid defensive secretion. Distinguished from these three genera by having head+19 rings *vs.* head+20 rings body plan, and pore formula 5+7-18 *vs.* 5+7+9-19 in *Victoriombrus* and the normal pore formula in *Atalopharetra* and *Bromodesmus*. Similar to *Noteremus*, *Paredrodesmus* and *Procophorella* in head+19 rings, pore formula 5+7-18 and a trapezoidal array of spinnerets; distinguished from these three genera in having sphaerotrichomes on male legs.

##### Description.

As for the type species.

##### Name.

Anagram of ‘Catherine’, for the millipede specialist Catherine Car, collector of the type specimens; gender feminine.

#### Cernethia inopinata

Taxon classificationAnimaliaPolydesmidaDalodesmidae

Mesibov sp. n.

http://zoobank.org/C4B48F32-BB9A-4C24-9B84-BC363F4C75D0

**Figure 1.**
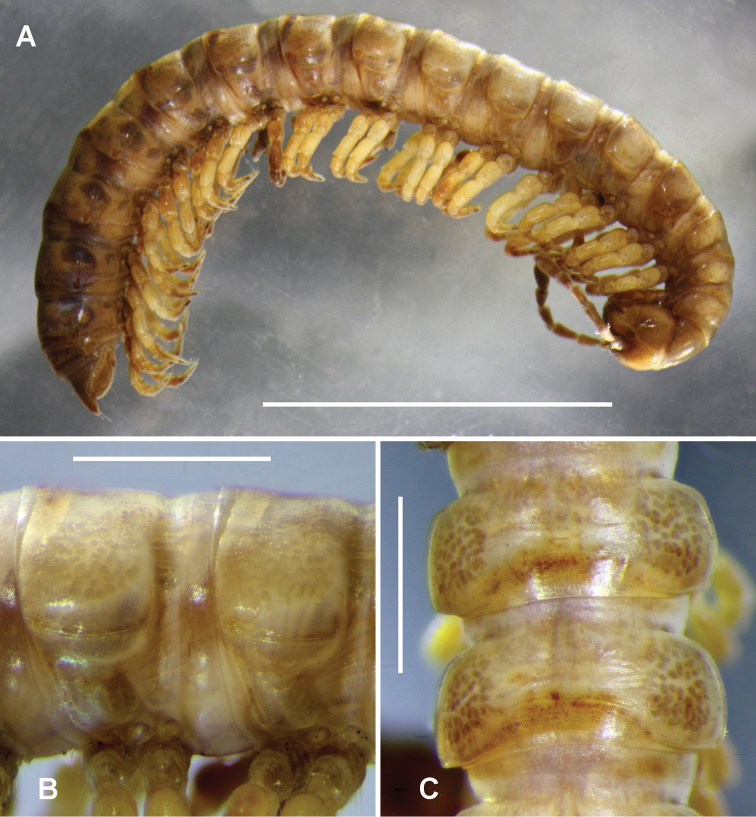
*Cernethia inopinata* sp. n., male paratype ex AM KS.114908. **A** Habitus **B** Midbody rings, right lateral view **C** Midbody rings, dorsal view. Scale bars: 5 mm (**A**); 1 mm (**B, C**).

**Figure 2.**
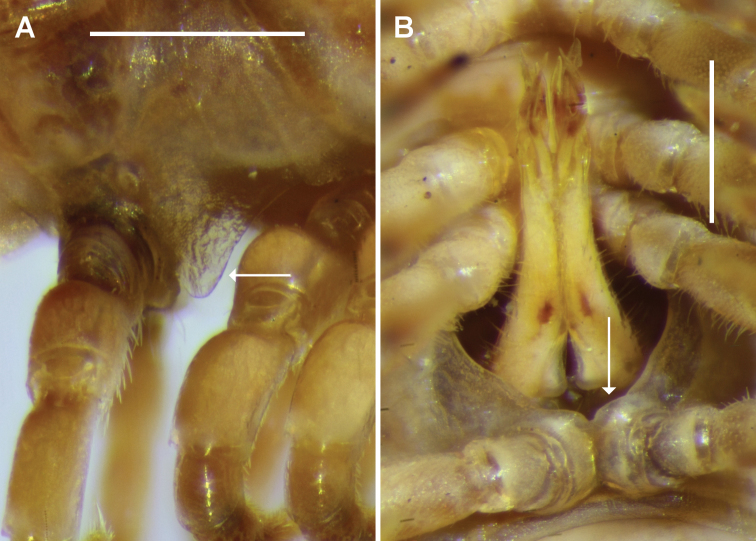
*Cernethia inopinata* sp. n., male paratype ex AM KS.114908. **A** Aperture on ring 7, right lateral view; posterolateral rim marked with arrow **B** Gonopods in situ, ventral view; note sternite (arrow) overlapping rear of aperture. Scale bars: 0.5 mm.

**Figure 3.**
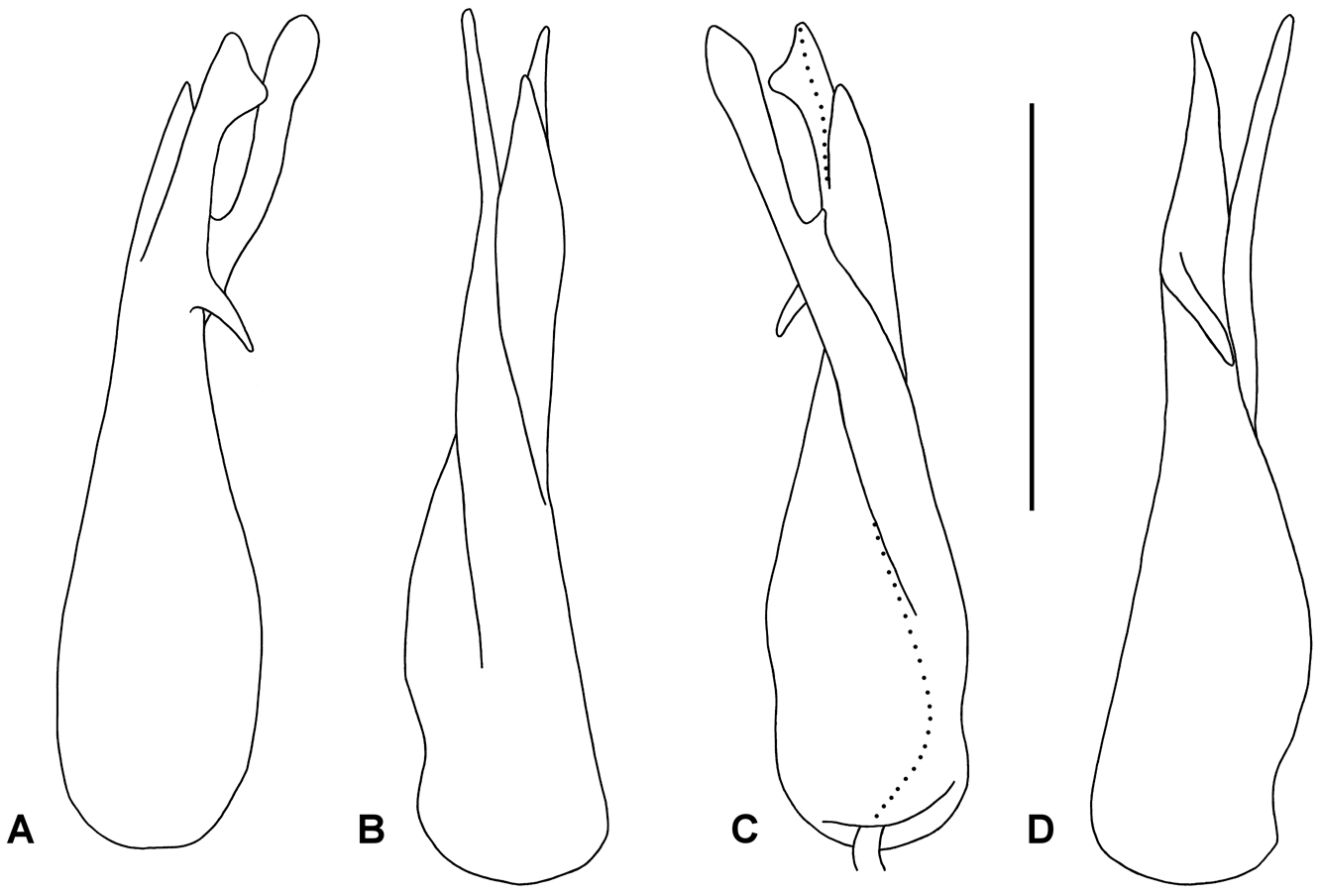
*Cernethia inopinata* sp. n., male paratype ex AM KS.114909. **A** lateral, **B** anterior, **C** medial and **D** posterior and slightly lateral views of right gonopod telopodite. Setation not shown; dotted line indicates course of prostatic groove. Scale bar: 0.5 mm.

**Figure 4.**
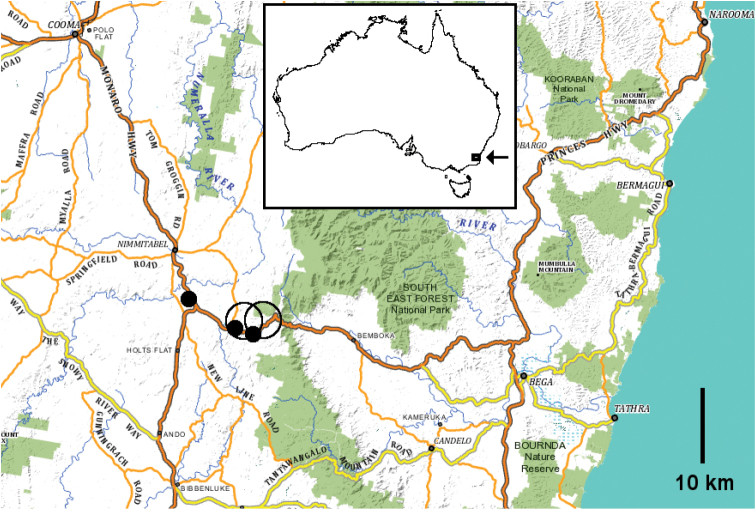
Known localities of *Cernethia inopinata* sp. n. as of 22 June 2015; black dots are for accurately located AM samples, black circles are for approximately located ANIC samples. Base map from the Spatial Information Exchange, New South Wales Department of Finance and Services (http://maps.six.nsw.gov.au/; accessed 14 June 2015). Inset shows location of main map (arrow) on map of Australia.

Figs 1 , 2 , 3 , map Fig. 4

##### Holotype.

Male, Glenbog State Forest near junction of Steeple Flat Road and Snowy Mountains Highway, ca. 10 km SE of Nimmitabel, NSW, -36.6128 149.3686 ±100 m, 1160 m a.s.l., 17 May 2006, C.A. Car, transect site T21, AM KS.124129 (ex AM KS.114908).

##### Paratypes.

**AM**: 5 males, 7 females, 10 juveniles, details as for holotype, KS.114908; 2 males, 3 females, same details but 19 September 2006, KS.114909; 6 males (1 male missing head + rings 2-5), 2 females, 6 juveniles, same details but 29 March 2006, C.A. Car, KS.94875.

##### Other material.

**AM**: 1 male, ca. 5 km SE of Nimmitabel at Bombala turnoff, NSW, -36.5806 149.3153 ±100 m, 1100 m a.s.l., 14 February 2007, C.A. Car, transect site T20, under bark of fallen log in wattle grove, KS.114919; 1 male, ca. 50 km W of Bega, Brown Mountain, Pipers Lookout, Monterey Road, NSW, -36.6203 149.4033 ±100 m, 1100 m a.s.l., 16 February 2007, C.A. Car, transect site T22, under bark scraps on ground, KS.114912; 3 stadium 6 juveniles, same details, KS.114913. **ANIC**: 3 males, Brown Mountain, NSW, -36.6 149.3833 ±2 km, 5 January 1967, R.W. Taylor, ANIC berlesate no. 9, rainforest, 64-000344; 1 male, same locality but 9 December 1967, R. Taylor and J. Brooks, ANIC berlesate no. 41, leafmould, 64-000345; 5 males, 2 females, same details but ANIC berlesate no. 42, leafmould, 3000 feet, 64-000346; 2 males, same details but ANIC berlesate no. 42C, 64-000347; 1 male, Rutherford Creek, Brown Mountain, NSW, -36.6 149.4167 ±2 km, 9 January 1968, M. Upton, ANIC berlesate 55, rainforest, leafmould, 64-000348; 2 males, same locality but 15 January 1969, S.R. Curtis, ANIC berlesate no. 129, 64-000349; 8 males, same locality but 26 May 1970, R.W. Taylor and R. Bartell, ANIC berlesate 287, rainforest, 64-000350.

##### Description.

Male and female adults with head+19 rings (Fig. 1A). Male/female approximate measurements: length 16/14 mm, maximum width across paranota 1.7/1.9 mm, maximum prozonite width 1.3/1.7 mm. In alcohol, well-coloured specimens yellowish brown with faintly reddish brown antennae, distal podomeres and lateral paranotal margins; some specimens with larger reddish-brown patches elsewhere, but patterning inconsistent.

Male with vertex of head bare, frons and clypeus sparsely setose; vertigial sulcus extending ventrally ca. halfway to line joining antennal socket centres; postantennal groove narrow, slightly impressed; antennal sockets separated by about 1.5× socket diameter. Antenna clavate, reaching dorsally to rear of tergite 2; relative antennomere lengths 6>3>2>(4=5), antennomere 6 widest. Collum half-moon-shaped in dorsal outline, anterior margin straight, posterior corner rounded. Relative overall ring widths collum<(head=2,3)<4<(5-15 equal)>16>17>18. Waist on diplosegments shallow, without striations; prozonites and metazonites smooth; metazonites with three transverse rows of sparse, very short, fine setae, mainly missing; limbus with widely spaced, narrow, pointed elements. Ring 2 paranotum with lateral margin straight, lower than collum and ring 3 paranotal margin. Midbody paranota (Figs 1B, 1C) with anterior corners widely rounded, posterior corners narrowly rounded and not extending posteriorly; lateral margins more or less horizontal, at ca. 1/2 ring height. Paranota greatly reduced on rings 16 and 17, not detectable on 18. Midbody sternites moderately setose, longer than wide; impressions well developed, longitudinal deeper than transverse. Ozopore small, round, opening dorsolaterally at posterior corner of paranotum; pore formula 5+7-18. Spiracle openings small, round; on diplosegments with anterior spiracle dorsal to anterior leg and posterior spiracle midway between legs and dorsal to leg bases. Midbody legs short, relative podomere lengths (femur=tarsus)>prefemur>(postfemur=tibia). Prefemur dorsally swollen on anterior legs except 1 and 2; femur slightly swollen dorsally on anterior legs. Sphaerotrichomes on all legs except 1 and 2, more numerous anteriorly, mainly on tarsus and tibia, anteriorly also on postfemur; sphaerotrichomes with shafts tapering to fine point, on midbody legs directed at ca. 45° to long leg axis, shorter and more erect anteriorly. Brush setae unbranched, slightly tapered with blunt tips, on all legs except 1 and 2, anteriorly on coxa, prefemur, femur and postfemur, denser anteriorly. Pre-anal ring very sparsely setose; hypoproct paraboloid; epiproct extending well past anal valves, tip truncate and slightly emarginate; spinnerets in trapezoidal array, ventral spinnerets slightly further apart than dorsal.

Gonopore on short, truncate cone arising distomedially on leg 2 coxa. Leg 6 and 7 bases equally widely separated by shallow concavity, leg 5 bases more narrowly separated; small tab on sternite close to each leg 6 base. Aperture 1/3-1/2 width of ring 7 prozonite, rim posterolaterally greatly extended ventrally (Fig. 2A), sternite between legs 9 overlapping posterior margin of aperture (Fig. 2B). Gonopod telopodites more or less straight (Fig. 2B), when retracted reaching space between leg 5 bases.

Gonocoxae short, truncated-conical, incompletely fused anteriorly in syncoxite, sparse setae on posterolateral and anteromedial surfaces. Cannula swollen at base, tapering abruptly and looping tightly to enter base of telopodite towards medial side. Telopodite (Figs 2B, 3) with cylindrical base, tapering from ca. 1/4 telopodite height, the main portion divided at ca. 3/4 telopodite height into two closely appressed processes: mediolaterally flattened solenomere with rounded tip and rounded subapical projection on posterior margin, and bluntly acuminate anteromedial process, slightly shorter than solenomere. Just basal to this telopodite division (i.e., just under 3/4 telopodite height), a small, finger-like, blunt process arising on posterolateral telopodite surface curving basally and posteromedially. Large, more or less flat process arising anteromedially, closely appressed to medial telopodite surface, directed distally and slightly posteriorly, curving slightly laterally to lie just medial and posterior to solenomere; apex laminar, slightly expanded with broadly rounded distal margin, terminating at about solenomere height; small tooth on anterior edge of process at ca. 2/3 process length (ca. 3/4 telopodite height). Sparse, long setae on posterior telopodite surface from base to just basal to level of short, finger-like process. Prostatic groove running more or less straight to tip of solenomere, posterior to base of large medial process.

Female more robust than male but shorter, legs thinner and without swellings, paranota not as well developed. Epigynum 1/3–1/2 ring 2 width, slightly raised medially; cyphopods not examined.

##### Distribution.

So far known only from high-elevation, open eucalypt forest and rainforest in the southeastern corner of the Monaro Tablelands in New South Wales (Fig. 4). The climate in the *Cernethia inopinata* sp. n. range is cool temperate but fairly dry, with a mean annual rainfall at nearby Nimmitabel (1075 m a.s.l.) of 687 mm, well-distributed through the year, and mean temperature minima and maxima of ca. -1.5 °C and 8 °C in June and July and ca. 9 °C and 22 °C in January and February (http://www.bom.gov.au/climate/averages/tables/cw_070067.shtml; accessed 13 June 2015). Snow patches lie on the ground in the open forest in the winter months.

##### Name.

Latin *inopinatus*, ‘unexpected’, for the unexpected presence of sphaerotrichomes (see Introduction).

##### Remarks.

The gonopod in *Cernethia inopinata* sp. n. resembles the gonopods of some species of *Gephyrodesmus* Jeekel, 1983 and *Orthorhachis* Jeekel, 1985 (Mesibov 2008) in having two large, closely appressed branches at the end of a strongly tapering telopodite. In all species of *Orthorhachis* the major branching occurs at more than half the telopodite height, in *Cernethia inopinata* sp. n. and all species of *Gephyrodesmus* at one-third or less the telopodite height. *Cernethia inopinata* sp. n. differs from *Gephyrodesmus* species in having an apically divided solenomere, as opposed to an undivided one. *Gephyrodesmus* and *Orthorhachis* species also differ from *Cernethia inopinata* sp. n. in having a head+20 rings body plan, wide paranota and a square spinnerets array.

The type specimens of *Cernethia inopinata* sp. n. were first examined in 2007, not long after they were collected. The samples smelled strongly at the time with an odour similar to that of the acrid defensive secretions of Tasmanian *Atalopharetra* and *Bromodesmus* species. The samples have had several changes of alcohol since 2007 and no longer have a strong odour.

## Supplementary Material

XML Treatment for Cernethia

XML Treatment for Cernethia inopinata
